# Circadian disruption induced by light-at-night accelerates aging and promotes tumorigenesis in young but not in old rats

**DOI:** 10.18632/aging.100120

**Published:** 2010-03-04

**Authors:** Irina A. Vinogradova, Vladimir N. Anisimov, Andrey V. Bukalev, Viktor A. Ilyukha, Evgeniy A. Khizhkin, Tatiana A. Lotosh, Anna V. Semenchenko, Mark A. Zabezhinski

**Affiliations:** ^1^ Petrozavodsk State University, Petrozavodsk 185910, Russia; ^2^ Department of Carcinogenesis and Oncogerontology, N.N.Petrov Research Institute of Oncology, St. Petersburg 197758, Russia; ^3^ Institute of Biology, Karelian Research Centre, Russian Academy of Sciences, Petrozavodsk 185610; Russia

**Keywords:** Light-at-night, life span, tumorigenesis, rats

## Abstract

We evaluated the effect of exposure to constant light started at the age of 1 month and at the age of 14 months on the survival, life span, tumorigenesis and age-related dynamics of antioxidant enzymes activity in various organs in comparison to the rats maintained at the standard (12:12 light/dark) light/dark regimen. We found that exposure to constant light started at the age of 1 month accelerated spontaneous tumorigenesis and shortened life span both in male and female rats as compared to the standard regimen. At the same time, the exposure to constant light started at the age of 14 months failed to influence survival of male and female rats. While delaying tumors in males, constant light accelerated tumors in females. We conclude that circadian disruption induced by light-at-night started at the age of 1 month accelerates aging and promotes tumorigenesis in rats, however failed affect survival when started at the age of 14 months.

## Introduction

Light-at-night has become an increasing and essential part of modern lifestyle and leads to a number of health problems, including excess of body mass index, cardiovascular diseases, diabetes and cancer [1-10]. The International Agency for Research on Cancer (IARC) Working Group concluded that "shift-work that involves circadian disruption is probably carcinogenic to humans" (Group 2A) [11]. An increase in light pollution could be one of causes of the sharp rise of  mortality from breast cancer among Alaskan native peoples (Eskimo, Indian and Aleut) since 1969 [12]. It was shown that there is a significant positive correlationbetween geographical latitude and the incidence of breast, colon and endometrial carcinomas and absence of the correlation in a case of stomach and lung cancers [13].

According to the circadian disruption hypothesis, light-at-night might disrupt the endogenous circadian rhythm, and specifically suppress nocturnal production of pineal hormone melatonin and its secretion in the blood [9,10,14]. Earlier we have shown that the exposure to constant illumination started at the age of 1 months accelerated development of metabolic syndrome and spontaneous tumorigenesis, shortened life span in rats as compared to the standard (12 hours light/12 hours dark) regimen [15].  In this paper in the first time it was shown that the exposure to constant illumination started at the period of natural switching-off reproductive function has no effect or protective effect on antioxidant defense system, survival and tumorigenesis in rats.

## Results

### Effect of light/dark regimen on life span in rats

In male rats, the exposure to LL regimen started at the age of 1 month failed significantly influence the mean life span of all as well as the last of 10% survivors whereas the exposure to LL regimen started at the age of 14 months increased by 6.7% the mean life span (p>0.05), by 9.4% (p<0.01) the mean life span of the last 10% survivors and increases by 3 months the maximum life span of male rats (Table 1).

**Table 1.** Effect of the exposure to constant light started at the age of 1 month (LL-1) and at the age of 14 months (LL-14) on survival and life span in male rats. Notes: *  Number of rats at the age of 14 months. Difference with controls (LD) is significant:  a, p< 0.05; b, p< 0.01; #, in brackets 95% confidential  intervals. MRDT, mortality rate doubling time.

Parameters	Light/dark regimen		
LD	LL-1	LL-14
Number of rats*	43	34	90
Mean life span, days	766 ± 25.4	744 ± 28.0	818 ± 18.1 (+ 6.7%)
Maximum life span, days	1045	1005	1141
Mean life span of last 10% survivors, days	994 ± 9.2	1002 ± 1.8	1087 ± 8.3 (+ 9.4%)^b^
α x 10^3^, days^-1^	7.49 (7.20; 7.75)#	7.07 (6.90; 7.16)^a^	6.58 (6.28; 6.82)^a^
MRDT, days	92.6 (89.4; 96.3)	98.1 (96.9; 100.4)^a^	105.3 (101.6; 110.4.)^a^

**Table 2.** Effect of the exposure to constant light started at the age of 1 month (LL-1) and at the age of 14 months (LL-14) on survival and life span in female rats. Notes: * Number of rats at the age of 14 months. Difference with controls (LD) is significant: a, p<0.05; b, p<0.01; #, in brackets 95% confidential  intervals. MRDT, mortality rate doubling time.

Parameters	Light/dark regimen		
LD	LL-1	LL-14
Number of rats*	30	36	71
Mean life span, days	844 ± 33.6	658 ± 22.8^b^ (- 22.0%)	811 ± 20.0
Maximum life span, days	1167	956	1198
Mean life span of last 10% survivors, days	1129 ± 18.9	921 ± 19.7 (-18.4%)	1113 ± 24.9
α x 10^3^, days^-1^	5.74 (5.56; 6.01)	4.19 (4.01; 4.38)^a^	6.03 (5.79; 6.35)
MRDT, days	120.7 (115.3; 124.6)	165.6 (158.4; 173.1)^a^	114.9 (109.1; 119.6)

**Figure 1.**
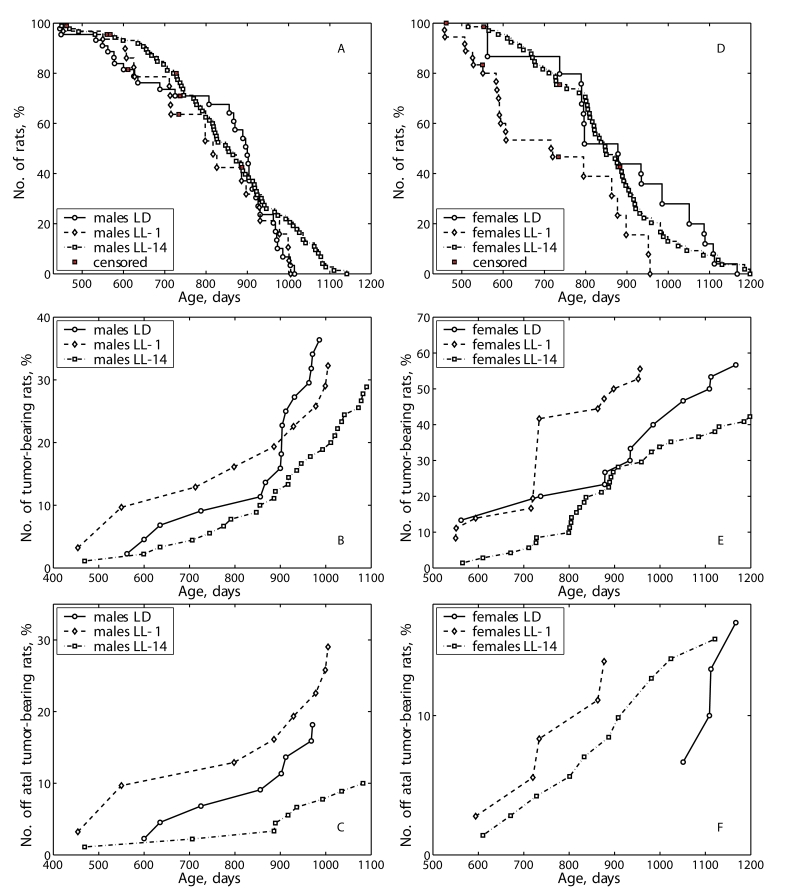
Effect of the exposure to various light regimens on tumorigenesis and survival in rats. (**A**) - survival, males;  (**B**) - total tumor incidence, males; (**C**) - fatal tumor incidence, males; (**D**) - survival, females; (**E**) - total tumor incidence, females; (**F**) - fatal tumor incidence, females.

At the same time, the rate of population aging (parameter α in the Gompertz equation) was slightly  decreased in LL-1 and in LL-14 groups as compared with the LD group males. The survival curve for males of the group LL-1  was significantly shifted to left in comparison to the survival curve for the group LD (Figure 1A) whereas was not in LL-14 group (Figure 1A).

In female rats, the exposure tothe LL regimen significantly decreased the mean life span (by 22.0%) and the population aging rate (by 27.0%) when started at the age of 1 month and failed to change both the mean life span and the aging rate when it was started at the age of 14 months (Table 2). The survival curve for females of the group LL-1  was significantly shifted to left in comparison to the survival curve for the group LD  whereas was not in LL-14 group (Figure 1D).

According to the log-rank test the conditional life span distributions of rats (given the animals survived the age of 14 months) kept under alternating day/night and two constant light regimens starting from one and 14 months of age differ insignificantly for males (p-value is 1.58E-01, χ2=3.7 on 2 df) and significantly for females (p-value is 6.31E-04, χ2=14.7 on 2 df). The difference between two groups of male rats kept under constant light regimens (LL-1 and LL-14) is significant (p-value is 1.02E-01, χ2=2.7 on 1 df). The life span distribution of females kept under constant light from the age of one month differs significantly from the control LD group (p-value is 1.39E-03, χ2=10.2 on 1 df) and from the group subjected to the constant light from the 14th month (p-value is 1.26E-03, χ2=10.4 on 1 df).

According to the estimated parameters of the Cox's regression model in males the constant light from older age decreases the relative risk of death compared to the group kept under the same regiment from earlier in life. Among the females, the LL-1 regimen increases the risk of death compared to the control group and the LL-14 decreases the risk of death compared to the LL-1 group (Table 3).

### Effect of light/dark regimen on spontaneous tumorigenesis  in rats

Pathomorphological analysis shows that benign tumors were most frequent in all groups of males and females. The significant part of them was represented by testicular Leydig cell tumors in males and mammary fibroadenomas in females (Tables 4 and 5).  Among malignant tumors lymphomas were most common however some cases of hepatocellular carcinoma, soft tissues sarcomas and sporadic carcinomas of other organs were detected.

The exposure to the LL-1 regimen accelerated sponta- neous tumors development as compared to the LD group and not influenced their total incidence both in male and female rats (Tables 4 and 5; Figure 1B and 1E). The first tumor in males of the LL-1 group was detected 5 months earlier than the first tumor in the LD group. The exposure to the LL-14 regimen did not influence the  incidence of spontaneous tumors in male and female rats.

**Table 3.** Cox's regression model parameters for experimental groups.

All rats	β	exp(β)	se(β)	p
Males LL-1 and LL-14	-0.41	0.67	0.25	1.00E-01
Females LD and LL-1	1.02	2.78	0.34	2.30E-03
Females LL-1 and LL-14	-0.82	0.44	0.26	1.70E-03

According to the log rank test the difference in life span distributions among all three groups of male rats with fatal and non-fatal tumors is significant (p-value is 4.85E-02, χ2=6.1 on 2 df). The pair-vise difference between LD and LL-1 groups is insignificant; between LD and LD-14 is significant (p-value is 3.32E-02, χ2=4.5 on 1 df); between LL-1 and LL-14 can be considered as significant (p-value is 1.10E-01, χ2=2.6 on 1 df). There was no significant difference in life span distributions among the female tumor-bearing rats.

According to the Cox's regression model the risk of death among the tumor-bearing male rats subjected to the LL-14 regiment is significantly lower compared to the LD group (β = -0.75; exp(β) = 0.47;  se(β) =0.36; p =  3.60E-02).

According to the log rank test there is no significant difference in life span distributions among male rats with fatal tumors subjected to different regiments. In females with fatal tumors the difference is significant among all three groups of rats (p-value is 8.30E-03, χ2=9.6 on 2 df); between LD and LL-1 groups (p-value is 4.50E-03, χ2=8.1 on 1 df) and between LD and LL-14 groups (p-value is 1.91E-02, χ2=5.5 on 1 df).

As estimated with the Cox's regression model the risk of death among female LD-14 rats with fatal tumors is sig-nificantly greater than for female rats under LD regiment (β = 1.36; exp(β) = 3.89;  se(β) = 0.62; p = 2.80E-02).

**Table 4.** Effect of the exposure to constant light started at the age of 1 month (LL-1) and at the age of 14 months (LL-14) on tumorigenesis in male rats. Notes: TBR - tumor-bearing rats.

Parameters	Light/dark regimen		
LD	LL-1	LL-14
Number of rats	43	34	90
Number of TBR (%)	15 (34.9%)	12 (35.3%)	26 (28.9%)
Number of malignant TBR (%)	8 (18.6%)	10 (29.4%)	9 (10%)
Total number of tumors	21	13	34
Number of tumors per TBR	1.40	1.08	1.31
Age at the time of the 1^st^ tumor detections, days	600	428	469
Mean life span of TBR, days	849 ± 34.7	786 ± 65.3	897 ± 32.1
Mean life span of fatal TBR, days	821 ± 52.2	794 ± 72.8	879 ± 62.5
*Localization and type of tumors*			
Testes: Leydigoma hemangioma	7	3	16
	1	-	1
Malignant lymphoma/ leukemia	3	6	3
Mammary gland: fibroadenoma	-	-	1
Liver: hepatocarcinoma	2	2	1
Skin carcinoma	-	-	1
Soft trissues: angiofibroma fibroma chondroma sarcoma malignant fibrous histoiocytoma	-	-	1
	-	-	1
	-	-	1
	-	-	4
	2	-	-
Lung: adenocarcinoma light-c ell carcinoma	-	1	-
	1	-	-
Small bowel: adenocarcinoma	-	1	-
Adrenal gland: cortical adenoma pheochromocytoma	3	-	3
	1	-	-
Urether: fibroma	1	-	-
Nervous system: paraganglioma	-	-	1
Total: benign malignant	13	3	25
	8	10	9

### Effect of light/dark regimen on free radical processes in rats

**Figure 2.**
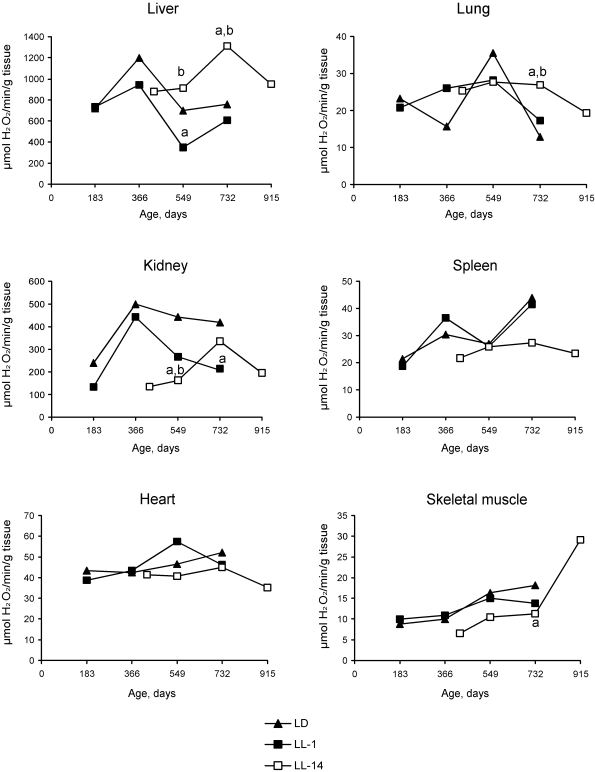
Effect of the exposure to various light regimens on age-related dynamics of the catalase activity in organs of rats. (**a**) - the difference with the relevant parameter in the group LD is significant, p<0.05; (**b**) - the difference with the relevant parameter in the group LL-1 is significant, p<0.05.

**Figure 3.**
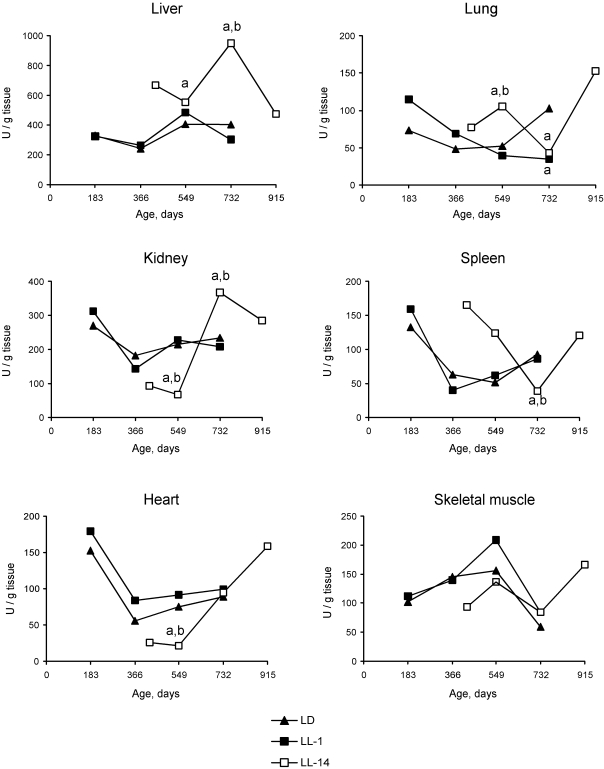
Effect of the exposure to various light regimens on age-related dynamics of the Cu,Zn-superoxide dysmutase (SOD) activity in organs of rats. (**a**) - the difference with the relevant parameter in the group LD is significant, p<0.05; (**b**) - the difference with the relevant parameter in the group LL-1 is significant, p<0.05.

Age-related changes in free radical processes should be generally described as desynchronization in activity of antioxidative enzymes and as a decreased antioxidant defense in the majority of organs. The changes of the functional activity of pineal gland induced by constant illumination affect both dynamics and level of  enzymatic activities. Most significant effects of  the age of start of the exposure to constant light on differences in the enzymatic activities were detected in the liver.  Thus, the activity of catalase revealed season cyclicity in rats of the group LD and LL-1.  In the group LL-14, the activity of both catalase and SOD was cyclic and revealed more high level as compared with the relevant parameters in the group LL-1.  Maximum levels of the enzymatic activity was detected at the age of 24 months  whereas in LD and LL-1 groups its where at the age of 12 and 18 months (Figures 2 and 3). There were age-related decrease in catalase activity in the groups LD and LL-1, but not in LL-14 group.

There were season changes  in dynamics of activity of antioxidant enzymes. Season variations in the activity of SOD were observed  in  heart, lungs and skeletal muscles, whereas  the activity of catalase - in kidney and skeletal muscles.  Age-related increase in catalase activity was observed in the skeletal muscles in rats of all three groups. The activity of SOD in lungs and spleen of rats in LL-14 group revealed U-shape curve pattern: it decreased at the age 24 months and increased at the age of 30 months. In the group LL-1 the decrease in SOD activity in lungs and spleen have been observed at the age of 12 months (Figures 2 and 3).

## Discussion

Our present data have shown that live-long maintenance of male and female rats at the  LL regimen started at the age of 1 month accelerated aging, decreased survival and promoted spontaneous tumorigenesis, whereas the exposure to constant illumination started at the age of 14 months  failed to reduce life span. Moreover it seems that LL-14 regimen had rather protective effect on survival and delayed age-related decrease in activity of antioxidant enzymes, SOD and catalase. Experiments in female rodents presented significantly evidence that exposure to constant illumination (24 hours per day) leads to disturbances in estrus function (persistent estrus syndrome, anovulation) [16-18] and spontaneous tumor development [1,17,19,20]. In all these studies the exposure to constant illumination has been started at the young adult age. There are evidences that the exposure to light at night time inhibits pineal production and secretion of melatonin - key pineal hormone [5,21,22]. It is worthy of note that old rodents are more susceptible to modifications of the photoperiod as compared with young ones [23]. In postmenopausal women, light at night suppressed serum melatonin level in higher degree then that in young cycling women. The exposure to constant illumination increases the lipid peroxidation in tissues and decreases both the total antioxidant activity and SOD activity, whereas treatment with melatonin inhibits lipid peroxidation, in the brain particularly [19,24-27].

**Table 5.** Effect of the exposure to constant light started at the age of 1 month (LL-1) and at the age of 14 months (LL-14) on tumorigenesis in female rats. Notes: TBR - tumor-bearing rats.

Parameters	Light/dark regimen		
LD	LL-1	LL-14
Number of rats	30	36	71
Number of TBR (%)	17 (56.7%)	20 (55.6%)	30 (45.3)
Number of malignant TBR (%)	1.47	1.75	1.37
Total number of tumors	5 (16.7%)	5 (13.9%)	11 (15.5%)
Number of tumors per TBR	25	35	41
Age at the time of the 1^st^ tumor detections, days	562	550	565
Mean life span of TBR, days	871 ± 51.4	732 ± 28.8	885 ± 29.3
Mean life span of fatal TBR, days	1098 ± 21.8	758 ± 52.0	868 ± 47.3
*Localization and type of tumors*			
Mammary gland: fibroma fibroadenoma adenocarcinoma	2	-	2
	11	19	25
	-	1	-
No of rats with benign mammary tumors	12 (40.0%)	16 (44.4%)	19 (26.8%)
Utery: polyp fibroma fibromyoma adenocarcinoma	2	4	-
	1	1	-
	-	1	-
	-	1	1
Skin: fibroma	-	-	1
Adrenal gland: cortical adenoma pheochromocytoma	1	3	2
	2	-	-
Ovary: fibroma	1	-	-
Pituitary: adenoma	-	1	-
Malignant lymphoma/ leukemia	3	2	7
Soft tissues: fibroma sarcoma	-	1	-
	2	1	2
Lung: adenocarcinoma	-	-	1
Total: benign malignant	20	30	30
	5	5	11

**Table 6.** Multifactor analysis of variance (MANOVA) evaluation of various factors effect on activity of antioxidant enzymes (Data represented as % of factors influences, *F*-ratio and  *p* value). Notes: Only significant data are presented. Empty columns means the absence of effect of a factor on enzyme activity.

**Organ**	**Factor**			
Age	Season	Light regimen	Time of the start of exposure to the constant light
**SOD activity**				
Liver			9.1% 11.01 0.0016	25.5% 26.02 0.0001
Heart		17.6% 6.67 0.0025		
Lungs		11.5% 3.90 0.026		
Skeletal muscle		11.7% 3.85 0.027		
**Catalase activity**				
Liver	9.1% 15.2 0.0003	30.8% 25.58 0.0001	20.1% 33.34 0.0001	32.2% 53.47 0.0001
Kidney		14.0% 7.84 0.001		
Skeletal muscle	23.0% 20.99 0.0001	8.0% 3.65 0.03		

Pierpaoli and Bulian [28] surgically pinealectomized BALB/c mice  at the age of 3, 5, 7, 9, 14 and 18 months and evaluated their life span. Results showed that while pinealectomy at the age of 3 or 5 months promoted acceleration of aging, no relevant effect of pinealectomy was observed when mice were pinealectomized at the age of 7 or 9 months. The remarkable life extension was observed when mice were pinealectomized at the age of 14 months. No effect was observed when the mice were pinealectomized at 18 months of age. The same aging-promoting or -delaying effects were confirmed in the hematological and hormonal-metabolic values measured. Evidence from the blood measurements showed that removal of the pineal gland in mice at the age of 14 months resulted in maintenance of more juvenile hormonal and metabolic patterns at 4th and 8th months after  pinealectomy  [28]. On the contrary,  a deleterious effect of pinealectomy was observed in mice subjected to the surgery at the age of 3 or 5 months. The authors suggest that the age of 14 months is the time when pineal gland accomplished its "aging program" and prevention of and/or recovery from aging becomes impossible. Our data on effect of "physiological pinealectomy" induced by the exposure to constant illumination started at the age of 1 or 14 on survival are in according with the observations of Pierpaoli and Bulian [28].  The results  of our experiments suggest that people at perimenopausal age could be  less susceptible to hazardous effect of constant illumination. This conclusion is not in contradiction with available data on age-related differences in susceptibility to carcino-genic agents is some tissues which were discussed earlier [29-31].

## Material and methods

Two hundred sixty seven male and 135 female outbreed LIO rats [32] were born during the first half of May, 2003. At the age of 25 days they were randomly subdivides into 4 groups (males and females separately) and kept at 2 different light/dark regimens: 1) standard alternating regimen (LD) - 12 hours light (750 lux): 12 hours dark; 2) constant light regimen (LL) -  24 hours light on (750 lux). At the age of 14 months the part of survived rats kept at the LD regimen were moved in the room with the constant light regimen (LL). Thus, the were 3 final groups: 1) LD; 2) LL-1 since the age of 1 months; 3) LL-14 since the age of 14 months. Only rats in each group survived the age of 14 months were included into protocols for calculations. The full data on the survival and tumorigenesis in control LD rats and in rats exposed to the LL since the age of 1 months have been presented elsewhere [15].

Some animals were sacrificed by decapitation, the appropriate tissues (liver, kidney, heart, lung, spleen and a skeletal muscle) dissected, weighed, and kept frozen at -25°С before carrying out of analyses. The samples of tissue of rats groups LD and LL-1 were collected at age 6, 12, 18 and 24 months, of the group LL-14 - at 14, 18, 24 and 30 months. Prior to enzyme determinations, thawed tissue samples were homogenized in 20 volumes of ice cold 50mM phosphate buffer (pH 7.4), centrifuged at 6000 g for 15 min at 5°C. The supernatant fraction was used for antioxidant enzyme determinations.

All animals were kept in the standard polypropylene cages at the temperature 21-23 ºC and were given ad libitum standard laboratory meal [33] and tap water. The study was carried out according to the recommendations of the Committee on Animal Research of Petrozavodsk State University about the humane treatment of animals.

The total SOD activity was measured using the epinephrine-adrenochrome reaction and was followed kinetically at 480 nm [34]. One unit of SOD was defined as the amount of enzyme required for 50% inhibition of the spontaneous epinephrine-adrenochrome transforma-tion. Catalase activity was measured by the method of Bears and Sizer [35] following the decrease in the absorption spectra of hydrogen peroxide at 240 nm caused by its decomposition by catalase. Activity of catalase defined as the amount of hydrogen peroxide in μmol that decomposed 1 g of tissue  per 1 minute.

All other rats were allowed to survive for natural death and were autopsied. Tumors as well as the tissues and organs with suspected tumor development were excised and fixed in 10% neutral formalin. After the routine histological processing the tissues were embedded into paraffin. 5-7μm thin histological sections were stained with hematoxylin and eosin and examined microscopically. Tumors were classified as fatal and non-fatal tumors and morphologically according to the IARC recommendations [36,37].

Experimental results were statistically processed by the methods of variation statistics and multifactor analysis of variance (MANOVA) with the use of STATGRAPH statistic program kit. The significance of the discrepancies was defined according to the Student t-criterion, Fischer exact method, χ2, non-parametric Wilcoxon-Mann-Whitney. Student-Newman-Keuls method was used for all pairwise multiple comparisons. Coefficient of correlation was estimated by Spearman method [38]. Differences in tumor incidence were evaluated by the Mantel-Haenszel log-rank test. Parameters of Gompertz model were estimated using maximum likelihood method, non-linear optimization procedure [39] and self-written code in 'Matlab'; confidence intervals for the parameters were obtained using the bootstrap method [40]. For experimental groups Cox regression model [41] was used to estimate relative risk of death and tumor development under the treatment compared to the control group: h(t, z) = h0(t) exp(zβ), where h(t,z) and h0(t) denote the conditional hazard and baseline hazard rates, respectively,  β is the unknown parameter for treatment group, and z takes values 0 and 1, being an indicator variable for two samples − the control and treatment group.

## Acknowledgments

The work was supported by grants from the President of the Russian Federation NSh-306.2008.4, and from the Russian Foundation for Basic Research 07-04-00546.
